# Neurosyphilis versus Herpes Encephalitis in a Patient with Confusion, Memory Loss, and T2-Weighted Mesiotemporal Hyperintensity

**DOI:** 10.1155/2012/154863

**Published:** 2012-02-16

**Authors:** Elisa Vedes, Ana Filipa Geraldo, Rita Rodrigues, Sofia Reimão, Alice Ribeiro, Francisco Antunes

**Affiliations:** ^1^Internal Medicine Department, Centro Hospitalar Lisboa Norte, EPE, 1649-035 Lisbon, Portugal; ^2^Neuroradiology Department, Centro Hospitalar Lisboa Norte, EPE, 1649-035 Lisbon, Portugal; ^3^Infectious Diseases Department, Centro Hospitalar Lisboa Norte, EPE, 1649-035 Lisbon, Portugal

## Abstract

Acute confusion and memory loss associated with asymmetrical mesiotemporal hyperintensity on T2-weighted MRI are characteristic of herpes encephalitis. The authors report the case of a patient with these symptoms and MRI presentation who had neurosyphilis. Recently clinical and imaging patterns usually associated with herpes simplex encephalitis have been seen in patients with neurosyphilis. Because syphilis is “The Great Pretender” not only clinically but also in imaging and because its numbers are rising, it must be sought as a differential diagnosis.

## 1. Introduction

Syphilis comes from the Greek word *syphlos,* meaning crippled or maimed. Since syphilis was first reported in 1941, its incidence has varied substantially. First it dropped because of the introduction of penicillin [1]. However, during the 80s, following increases in the rates of intravenous drug use and the number of people with multiple sexual partners, syphilis rates started rising. After 1990, with the emphasis on preventing human immunodeficiency virus (HIV) infection, syphilis incidence decreased in the USA and Western Europe but continued increasing in Eastern Europe, Central Asia, sub-Saharan Africa, and Latin America. Since the beginning of the new millennium the incidence is rising worldwide, but the elimination effort continues [2–4]. 

With the advent of neuroimaging and the increasing numbers of neurosyphilis some image patterns have been associated with this illness and clinicians must be alert to the differentials.

## 2. Case Presentation

 A 43-year-old black man, born in Angola, resident in Portugal, presented with a one-week history of confusion, memory loss with attention deficit, and strange behavior, being sometimes aggressive. He had no other symptoms. There was no relevant past medical history, no alcohol or drug abuse, and no recent travelling.

On admission, the patient had person, place, and time disorientation, as well as an ataxic gait. Mini-Mental State Examination score was 18/30 due to orientation, calculi, evocation, and language deficits. Kernig and Brudzinski signs were negative. The patient was afebrile hemodynamically stable and general physical and neurologic examinations were otherwise unremarkable. Blood test revealed only a slight rhabdomyolysis (CK 885 U/L) with no rise in acute phase inflammatory markers. Lumbar puncture was clear and revealed 26.4 cells/mm^3^, most of which lymphocytes, high protein count (92.9 mg/dL), and normal glycorrhachia.

**Figure 1:**
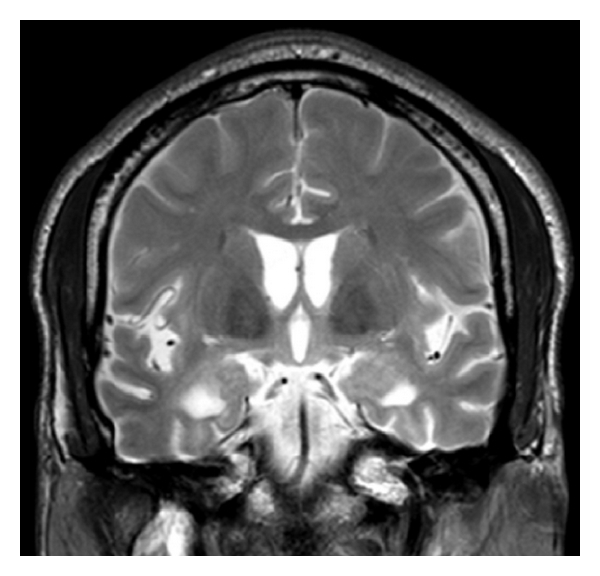
T2-weighted image on the coronal plane showing cortico-subcortical bilateral hyperintensity involving the mesiotemporal region, including amygdala and hippocampi with sulci and temporal horns enlargement.

**Figure 2:**
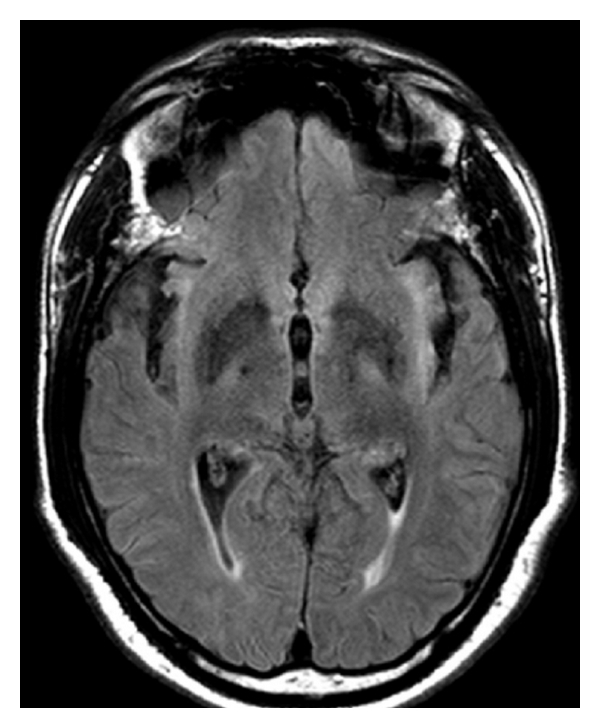
Axial FLAIR image depicting bilateral hyperintensity of the insula more prominent on the left.

Brain computed tomography (CT) scan showed a pattern of atrophy mainly involving the mesiotemporal region bilaterally, with focal enlargement of the lateral ventricles temporal horns. Magnetic resonance imaging was performed depicting better these atrophic changes, as well as an asymmetrical bilateral cortical and subcortical hyperintensity on T2- and fluid-attenuated-inversion-recovery- (FLAIR-) weighted signal in the mesiotemporal region and insula, more intense on the right side. There was no mass effect, restricted diffusion, or enhancement after the injection of gadolinium (Figures 1 and 2).

The patient was treated empirically with acyclovir for herpetic encephalitis. Rapid antibody testing for HIV1/2 was negative. Serologic testing for herpes simplex virus (HSV) 1 and 2 was positive for IgG antibodies, but no IgM antibodies were detected and cerebrospinal fluid (CSF) polymerase chain reaction (PCR) for HSV was negative. The results of the serum rapid plasma reagent (RPR) test (titer, 1 : 256) and serum *Treponema pallidum* haemagglutination (TPHA) test (titer, 1 : 20480) were positive. CSF TPHA titer was 1 : 10240 and Venereal Disease Research Laboratory test (VDRL) titer was 1 : 32. The positive CSF VDRL established the diagnosis of neurosyphilis, and the high negative predictive value of a negative PCR for HSV ruled out herpetic encephalitis. The patient received a 14-day course of penicillin with a minor cognitive improvement. When the wife was approached, she remembered the patient had a penile lesion eleven years before the admission that he only “treated” with cream.

Brain MRI was repeated immediately after the antibiotherapy and showed no improvement of the imaging findings. On the contrary, there was a discrete extension of the high signal on T2 and FLAIR sequences in the left mesiotemporal region and insula.

The patient was discharged and continues follow-up at the Infectious Diseases Clinic.

## 3. Discussion

When Sir William Osler said “He who knows syphilis, knows medicine”, was referring to the wide range of clinical manifestations associated with this disease. As tuberculosis, syphilis is known as “The Great Pretender.” With the advent of neuroimaging, this designation appears to apply also to the imaging presentation of the disease.

Meningovascular involvement is the most frequent pattern of neurosyphilis. On neuroimaging studies, this kind of neurosyphilis may present as infarction, leptomeningeal enhancement, arteritis, cerebral *gummata*, meningoneuritis with cranial nerve palsies or labyrinthitis. In other kind of central nervous system (CNS) syphilitic infection, named general paresis, MRI findings include cortical atrophy and areas of abnormal signal intensity [5–8]. 

Although asymmetric bilateral mesiotemporal T2 hyperintensity strongly suggests herpetic encephalitis, other diagnoses may be considered [9]. These include paraneoplastic limbic encephalopathy, acute hemorrhagic leukoencephalitis (Hurst's disease), gliomatosis cerebri, systemic lupus erythematosus, primary CNS lymphoma, and complex partial status epilepticus [5, 10]. Rarely, this imaging pattern has also been associated with neurosyphilis [5, 10–14]. The cause of the signal hyperintensity in neurosyphilis is not well known, but some authors believe it is a consequence of edema and gliosis [5]. 

Some imaging features may help to differentiate between this rare presentation of neurosyphilis and the more common HSV encephalitis. The former usually presents with atrophy of the medial temporal lobe rather than the mass effect caused by cortical and subcortical edema associated with the latter [5]. Atrophy is related with the indolent course of neurosyphilis, contrasting with the fulminant herpetic encephalitis [6]. On the other hand, gyral enhancement, signs of hemorrhage, or areas of restricted diffusion are typically absent in neurosyphilis but frequently described in HSV infection [9].

In this paper, not only the imaging features but also the clinical course was more characteristic of HSV encephalitis than of neurosyphilis as the patient had only a week long symptomatic course of confusion and loss of memory. Most commonly, syphilis clinical course is indolent and neurosyphilis causes no symptoms. When symptomatic, neurosyphilis frequently presents as a “stroke syndrome,” usually with prodromes like headaches, vertigo or insomnia. Accordingly, the patient was initially started on acyclovir and only after the serologies and CSF test results changed to penicillin.

The patient showed only minor cognitive improvement with bilateral mesiotemporal abnormalities persistence immediately after the therapy. Some reports describe an improvement of the signal hyperintensity weeks or months after the completion of the treatment [5, 10]. However, these lesions may become irreversible in late stage disease and medial temporal atrophy involving the hippocampus has been considered a poor prognostic sign [7, 15]. 

The present case emphasizes that neurosyphilis may mimic not only the clinical pattern but also the neuroimaging aspects of herpetic encephalitis. Thus, it is important to remember that patients with this kind of presentation may have syphilis and this must be checked out and treated.
